# Polydopamine Functionalization of Aligned Polylactic Acid Microfibers for Enhanced Neural Cell Interaction and Oxidative Stress Mitigation

**DOI:** 10.1021/acsomega.6c07293

**Published:** 2026-10-28

**Authors:** Francisco Navarro-Páez, Fernando Gisbert Roca, Manuel Monleón Pradas

**Affiliations:** † Center for Biomaterials and Tissue Engineering, 16774 Universitat Politècnica de València, C. de Vera s/n, Valencia 46022, Spain; ‡ Networking Research Center on Bioengineering, Biomaterials and Nanomedicine, Valencia 46022, Spain

## Abstract

Peripheral nerve injuries (PNIs) result in severe functional impairments and pose a significant clinical and socio-economic burden. Although autologous nerve grafts remain the clinical gold standard, their use is limited by donor availability and associated morbidity. Biomaterial-based strategies incorporating aligned architectures have emerged as promising approaches to provide directional guidance for regenerating axons. However, synthetic polymers commonly used for these systems lack intrinsic bioactivity and offer limited protection against the oxidative microenvironment present after injury. Here, aligned polylactic acid (PLA) microfibers were functionalized with polydopamine (PDA) via a one-step immersion process to introduce bioactive and antioxidant surface properties. The PDA-coated microfibers were systematically characterized in terms of morphology, surface chemistry, roughness, antioxidant capacity, and hydrogen peroxide scavenging. In vitro assays demonstrated that PDA functionalization enhanced Schwann cell attachment and improved cellular tolerance to hydrogen peroxide-induced oxidative stress, while also enhancing dorsal root ganglion adhesion and neurite outgrowth along the microfiber axis. Overall, these results indicate that PDA functionalization enhances the biological performance of aligned PLA microfibers by integrating structural guidance with oxidative stress modulation, highlighting their potential as versatile guidance elements for future nerve repair applications.

## Graphical abstract

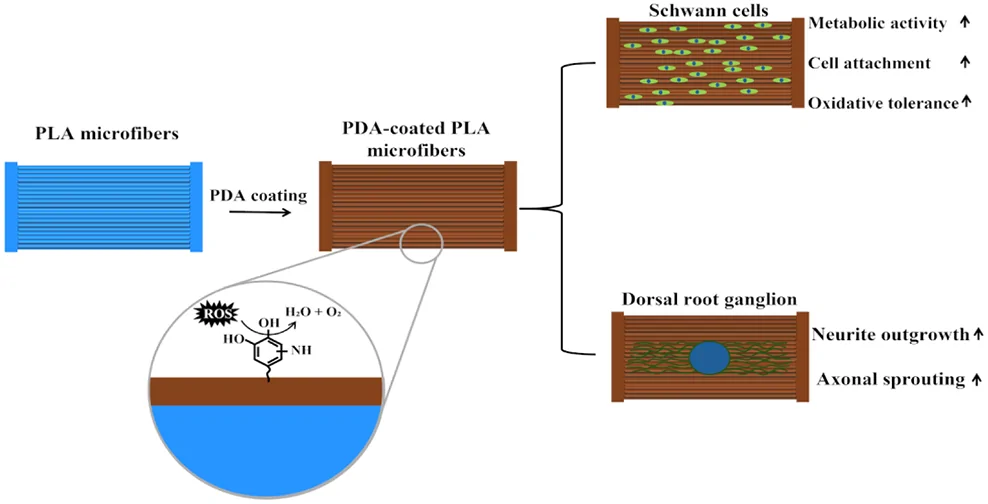

## 1 Introduction

Peripheral nerve injuries (PNIs) impose a substantial medical and socio-economic burden and are often associated with long-term impairment and reduced quality of life.^1,2^ Autologous nerve grafts remain the gold standard for critical nerve defects.^3^ However, their use is constrained by limited donor tissue availability and donor-site morbidity, among other factors.^1,4^ As a result, tissue engineering approaches have been widely explored to develop alternative strategies for peripheral nerve repair. Despite significant progress, complete functional recovery following PNI remains elusive.^5^ These limitations highlight the need for regenerative platforms capable of providing both structural and biological cues to support nerve regeneration.

Among these strategies, the introduction of aligned guidance architecture has emerged as a promising approach to direct axonal growth and improve regenerative outcomes. The orientation of these microfibers can provide contact guidance, influence cell alignment and direct neurite extension along a defined path.^6–9^ A wide range of polymers can be used to fabricate microfibers; among them, polylactic acid (PLA), a biocompatible and biodegradable synthetic polymer, has gained increasing attention in recent years^10,11^ and even some PLA-based products have been approved by both the U.S. Food and Drug Administration (FDA)^12^ and the European Medicines Agency (EMA).^13^ Owing to its versatility, PLA has been employed to produce scaffolds with well-defined architectures, including aligned microfibers capable of providing topographical cues that guide cellular orientation and axonal growth.^14–16^

Despite these advantages, the intrinsic bioinertness of PLA, together with its lack of cell-recognition motifs and limited surface reactivity, restricts its effectiveness in nerve repair applications. Several surface modification strategies have been proposed to address these limitations; however, many rely on complex processing steps or nonenvironmentally friendly reagents (chemical conjugation using wet chemistry) and costly equipment (plasma treatment), limiting their translational appeal.^17^

Polydopamine (PDA) has emerged as a versatile surface modification strategy capable of addressing these limitations of bioinert polymers through a simple one-step immersion process, enabling the introduction of bioactive functionalities onto otherwise inert substrates.^18^ PDA is formed via the self-polymerization of dopamine monomers under alkaline conditions,^19^ and exhibits good *in vivo* biocompatibility.^20^ The resulting coating presents a range of functional groups, including catechol, amine, imine and quinone moieties,^21^ which has driven its widespread adoption for substrate functionalization in tissue engineering. PDA has been extensively used as a platform for the immobilization of bioactive molecules, such as growth factors, via Michael addition or Schiff base reactions, thereby enhancing regenerative responses across multiple tissue types,^22–24^ including peripheral nerve regeneration,^25,26^ where PDA has been used to immobilize neurotrophic factors. In these approaches, PDA primarily functions as a surface modification and immobilization platform for additional bioactive components.

Beyond its established role as a functionalization and immobilization platform, PDA possesses intrinsic antioxidant activity through its ability to scavenge reactive oxygen species (ROS).^27^ ROS comprises a class of oxygen-derived, redox-active molecules, including free radicals such as hydroxyl radicals and nonradical species such as hydrogen peroxide.^28,29^ Under physiological conditions, ROS are continuously generated and act as secondary messengers involved in the regulation of essential cellular processes.^30^ However, excessive ROS accumulation, such as that occurring following tissue injury due to inflammatory cell activation, can disrupt redox homeostasis, leading to oxidative stress and irreversible tissue damage.^29,30^ In this context, exploiting the intrinsic ROS-scavenging activity of PDA, rather than using the coating primarily as an intermediate layer for the immobilization of additional bioactive agents, represents an attractive strategy for introducing antioxidant functionality into nerve-regeneration scaffolds. Importantly, such biochemical functionality should complement, rather than replace, the structural cues required to direct regenerating neurites.

In this study, we investigated whether the intrinsic antioxidant functionality of PDA could be integrated with the topographical guidance provided by aligned PLA microfibers without requiring additional bioactive agents. Two PDA coating durations (8 and 48 h) were evaluated to generate different extents of PDA deposition and to examine how coating-dependent physicochemical changes relate to antioxidant functionality and biological response. The PDA-functionalized microfibers were systematically characterized in terms of their morphological and physicochemical properties, antioxidant activity, and ability to support Schwann cell attachment, proliferation, and alignment. The biological relevance of the antioxidant functionality was further investigated by evaluating Schwann cell responses under H_2_O_2_-induced oxidative stress. Finally, DRG explants were used to determine whether PDA functionalization could enhance neurite outgrowth while preserving the directional guidance provided by the aligned microfiber architecture. Through this approach, we aimed to establish the relationship between PDA functionalization, antioxidant activity, and neural cell response within an aligned guidance platform, thereby assessing its potential as a guidance element for peripheral nerve regeneration.

## 2 Materials and Methods

### 2.1 Polylactic Acid Microfiber Preparation

The aligned PLA microfiber bundles were obtained by grouping 1000 extruded PLA microfibers with a diameter of 10 μm (Aitex Textile Research Institute). The ends of the fiber bundles were thermally fused (Fisherbrand Heat Sealer–15350360). PLA microfiber bundles with the same number of microfibers and length (10 mm) were used for every assay.

### 2.2 Polydopamine Coating

The direct-immersion method was used to deposit polydopamine onto the microfiber bundles. Dopamine hydrochloride (H8502, Sigma-Aldrich) was dissolved in 10 mM Tris-HCl buffer (pH 8.5; 252859, Sigma-Aldrich) to obtain a final concentration of 2 mg/mL and briefly stirred at 200 rpm for 1 min. The microfiber bundles were fully submerged in the dopamine solution and incubated under continuous shaking (100 rpm) at room temperature for either 8 or 48 h. After coating, all samples were rinsed three times for 5 min in Milli-Q water to remove nonadherent PDA. Up to this point, the procedure was identical for all samples. Subsequent handling differed according to the downstream assay: Samples intended for physicochemical characterization were washed three times in 70% ethanol (ET0002025S, Scharlau) for 10 min, followed by three washes in Milli-Q water, and then dried under vacuum at room temperature for 48 h. Samples intended for cell culture assays underwent the same washing sequence, but all steps were performed under sterile conditions. After the final sterile Milli-Q wash, samples were conditioned for 24 h before cell seeding instead of being vacuum-dried.

Coatings produced after 8 and 48 h of immersion are hereafter referred to as PLA–PDA 8 h and PLA–PDA 48 h, respectively. Uncoated PLA microfibers served as controls and are denoted PLA.

### 2.3 Morphological and Physicochemical Characterization of PDA-Coated Microfibers

#### 2.3.1 Macroscopic and Microscopic Imaging

Uncoated and coated microfiber bundles were first imaged with a stereomicroscope (MZ APO, Leica), then fiber orientation was assessed in ImageJ^31^ through the “Directionality” built-in plugin, applying the Fourier-based analysis (*n* = 5). The surface morphology of microfiber bundles was further characterized by high-resolution field-emission scanning electron microscopy (HRFESEM; ZEISS GeminiSEM 500). The samples were mounted onto a carbon tape and gold-sputter-coated, and micrographs were acquired at 1.5 kV accelerating voltage with a working distance of 4 mm.

#### 2.3.2 Atomic Force Microscopy (AFM)

AFM imaging and surface roughness measurements were performed in tapping mode (Multimode 8, Bruker) with antimony (n)-doped RTESPA probe (0.01–0.025 Ω·cm, MPP-21120–10). Scans (*n* = 5) were collected over a 5 μm^2^ area, and data processing (curvature removal and roughness analysis) was carried out using NanoScope Analysis software (version 1.8).

#### 2.3.3 Thermogravimetric Analysis (TGA)

Thermal degradation profiles of the coated samples (*n* = 3) were investigated using a TGA/SDTA851 thermogravimetric analyzer (Mettler-Toledo operated by the STARexx software). A heating ramp was applied from 30 to 800 °C at a heating rate of 10 °C/min with a nitrogen flux of 50 mL/min while the mass loss of the samples was monitored. As a result, thermograms in which the samples’ residual weight is represented as a function of temperature were obtained and the most representative thermogram of each sample was plotted.

#### 2.3.4 Fourier Transform Infrared Spectroscopy (FTIR) Analysis

To confirm the deposition of PDA on PLA microfiber bundles’ surface, Fourier transform infrared spectroscopy (Cary 630 FTIR spectrometer, Agilent Technologies) was performed in attenuated total reflection (ATR) mode. The spectra were obtained from averages of 24 scans at 4 cm^–1^ resolution, between 400 and 4000 cm^–1^. Under the same conditions, an air background measurement was performed and used as baseline correction. Three samples from each group were analyzed (*n* = 3), and the most representative spectrum is shown.

### 2.4 In Vitro ROS Assays

#### 2.4.1 ABTS^•+^ Radical Scavenging Assay

The total antioxidant capacity of PLA and PDA-coated PLA microfibers (8 and 48 h) was assessed using the 2,2-Azino-bis­(3-ethylbenzothiazoline-6-sulfonic acid) (ABTS; A1888, Merck) colorimetric assay. A 14 mM ABTS stock solution was converted to its radical cation form (ABTS^***•** ^**+**^*^) by mixing it 1:1 with a 4.9 mM potassium persulfate (K_2_S_2_O_8_; 216224, Merck) solution and incubating the mixture overnight at room temperature protected from light. The resulting *ABTS* ^•+^solution was diluted in Milli-Q water (1:70), and its absorbance at 734 nm was verified to be approximately 0.7.

PLA, PLA–PDA 8 h, and PLA–PDA 48 h microfiber bundles (*n* = 5) were incubated in the *ABTS* ^***•+***^ solution, and absorbance at 734 nm was recorded at multiple time points (0.5, 1, 1.5, 2, 3, 4, 5, and 6 h) using a microplate reader (Synergy LX Multi-Mode Microplate Reader, BioTek). The scavenging activity was calculated using formula (1):

$$Scavenging\left(\%\right)=\left(1-\frac{A_{sample}}{A_{control}}\right)\times 100$$

where *A*_sample_ is the absorbance of the *ABTS* ^•+^ solution in the presence of the fiber bundle, and *A*_control_ is the absorbance of the *ABTS* ^•+^ solution alone. Data were expressed as mean ± standard deviation (SD).

#### 2.4.2 Hydrogen Peroxide Scavenging

The ability of PLA and PDA-coated PLA microfibers (8 and 48 h) to scavenge hydrogen peroxide (H_2_O_2_) was evaluated following a modified version of the protocol described in ref 32. Microfiber bundles (*n* = 5) were placed in a nontreated 48-well culture plate and incubated with 150 μL PBS (pH 7.4, 21-031-CM, Corning) per well. Subsequently, 150 μL of a freshly prepared 150 μM H_2_O_2_ solution (H1009, Sigma-Aldrich) was added. After 1 h of incubation in the dark, the bundles were removed and 300 μL of 1 M potassium iodide (KI; PO04100250, Scharlau) was added to each well. After a 5 min reaction period, absorbance at 350 nm was measured with a microplate reader. A H_2_O_2_/KI solution (without fibers) was used as a control, and PBS/KI as the blank. The H_2_O_2_ scavenging activity was calculated as

$$\mathrm{Scavenging}\left(\%\right)=\left(\frac{A_{\mathrm{sample}}-A_{\mathrm{blank}}}{A_{\mathrm{control}}-A_{\mathrm{blank}}}\right)\times 100$$

where *A*_sample_ is the absorbance of the solution after incubation with fibers, *A*_control_ is the absorbance of the H_2_O_2_/KI solution, and *A*_blank_ corresponds to KI in PBS. Data were expressed as mean ± standard deviation (SD).

### 2.5 Cell Culture Assays

#### 2.5.1 Sample Sanitization and Conditioning

Microfiber bundles were sanitized following the procedure described in Section 2.2 Polydopamine Coating. After sanitization, samples were conditioned for 24 h in DMEM high glucose (4.5 g/L; 10313021, ThermoFisher Scientific) supplemented with 1% penicillin/streptomycin (15140122, ThermoFisher Scientific) at 37 °C and 5% CO_2_. After conditioning, the medium was removed and bundles were air-dried under sterile conditions for 30 min before cell seeding.

#### 2.5.2 Schwann Cell Culture on PDA–PLA Microfibers

Human Schwann cells (hSCs; P10351, Innoprot) were cultured on coated and uncoated microfiber bundles to assess their behavior. Cells were maintained in Schwann Cell Medium (P60123, Innoprot) at 37 °C in a humidified 5% CO_2_ atmosphere. After expansion in T75 flasks, passage-6 cells were detached with trypsin/EDTA (25200072, Gibco), centrifuged at 1080 rpm for 4 min, and seeded onto the samples at 5 × 10^4^ cells per fiber bundle. A 6 μL droplet containing the cell suspension was placed directly on each sample, which was then incubated for 15 min in a nontreated 24-well plate at 37 °C and 5% CO_2_. Subsequently, 100 μL of hSC medium was gently added to each well and the samples were left undisturbed to allow proper cell settling on the fibers. After an additional 30 min, 1 mL of culture medium was added. The cells were then maintained under the same incubation conditions, with medium changes performed every 2 days.

Cell viability was assessed on days 1, 3, and 7 using the PrestoBlue HS Cell Viability Reagent (P50200, Thermo Fisher). In brief, after culturing for 1, 3, and 7 days, the samples (*n* = 5) were transferred to a new nontreated P24 cell culture plate and washed with PBS and then incubated in 10% PrestoBlue solution in phenol red-free DMEM (31053028, Gibco). After 2 h of incubation at 37 °C and 5% CO_2_, three 100 μL aliquots from each well were transferred to a 96-well plate. Absorbance was recorded at 570 nm and adjusted using the corresponding 600 nm background reading. To account for background absorbance, blank wells containing the PrestoBlue solution and coated or uncoated fiber bundles, but no cells, were subtracted from all readings.

In addition, after 1 and 7 days of culture, immunostaining was performed to visualize hSCs morphology. Samples (*n* = 3) were washed with PBS and fixed with 4% paraformaldehyde (PFA; 47608, Sigma-Aldrich) for 15 min at room temperature (RT). Following fixation, the samples were washed three times with PBS for 10 min each. To prevent nonspecific binding and permeabilize the cell membrane, samples were immersed in a blocking buffer composed of 1 M phosphate buffer (PB; P3619, Sigma-Aldrich) diluted to 0.1 M with Milli-Q water and supplemented with 1% normal goat serum (NGS; 50062Z, ThermoFisher) and 0.1% Tween-20 (P1379, Sigma-Aldrich) for 1 h at RT. Samples were incubated overnight at 4 °C with SCs marker primary antibody, S100β (ab52642, Abcam), at a 1:200 dilution in blocking buffer. Following primary antibody incubation, the samples were washed three times with PBS and subsequently incubated with Alexa Fluor 488 goat anti-rabbit secondary antibody (A11008, Thermo Fisher) at a 1:200 dilution in blocking buffer for 2 h at RT. Cell nuclei were counterstained with DAPI. Finally, the samples were rinsed three times with PBS, mounted with Vectashield (Vector Laboratories), and visualized using a confocal microscope (Leica). Representative images acquired after 1 day of culture (*n* = 5 per sample) were analyzed with ImageJ software to obtain the nuclei count and Feret angle (percentages were calculated as frequency per bin divided by total nuclei). Analysis was restricted to the nuclei because the cytoskeletons of neighboring hSCs often overlapped, preventing reliable discrimination between individual cells.

#### 2.5.3 hSCs In Vitro ROS

##### 2.5.3.1 IC50

Before evaluating whether the ROS-scavenging capacity of PDA-coated fibers had biological relevance, the cytotoxic concentration range of H_2_O_2_ for hSCs was determined. At passage 6, hSCs were trypsinized and seeded in treated 96-well plates at a density of 2 × 10^4^ cells/well. Cells were allowed to attach for 24 h prior to treatment. A 1 mM H_2_O_2_ working stock was prepared in serum-free, phenol red-free DMEM and protected from light. From this stock, freshly prepared 2× dosing solutions were generated to achieve final concentrations of 0–1000 μM after 1:1 dilution with the medium already in the wells. All solutions were prepared immediately before use and kept on ice. Before exposure, the medium in each well (*n* = 5 per concentration) was removed, cells were washed three times with PBS and 50 μL fresh serum-free, phenol red-free DMEM was added. Then, 50 μL of the appropriate 2× H_2_O_2_ dosing solution was added and cells were incubated with H_2_O_2_ for 1 h at 37 °C. Control wells received medium without H_2_O_2_. After 1 h, the medium was aspirated, and three PBS washes were performed to ensure complete removal of residual H_2_O_2_, followed by the addition of fresh Schwann cell culture medium. Cells were allowed to recover for 24 h before viability assessment.

Cell viability was quantified using PrestoBlue. After washing cells three times with PBS, 100 μL 10% PrestoBlue working solution was added, and cells were incubated for 2 h at 37 °C, protected from light. Subsequently, the supernatant was transferred to a new plate and absorbance was recorded at 570 nm and adjusted using the corresponding 600 nm background reading. Blank wells containing the PrestoBlue working solution but no cells were subtracted from all readings. Wells treated with 0.1% Triton X-100 for 15 min served as death controls. Cell viability for each H_2_O_2_ concentration was calculated as

$$\mathrm{Viability}\left(\%\right)=\left(\frac{{\mathrm{Abs}}_{H_{2}O_{2}}}{{\mathrm{Abs}}_{\mathrm{Control}}}\right)\;\times \;100$$

where the absorbance values are blank corrected. Data were expressed as mean ± SD. The concentration–response curve was fitted using a four-parameter logistic model, and the IC_50_ value for H_2_O_2_ was extracted from the fitted curve.

##### 2.5.3.2 H_2_O_2_ Exposure on Seeded PLA vs PDA-Coated Microfibers

Schwann cell-seeded microfiber bundles (*n* = 5) were prepared as described in Sections 2.5.1 and 2.5.2 and cultured for 24 h before the assay. A 73 μM H_2_O_2_ solution (IC_50_ value) was freshly prepared using the dilution strategy described in Section 2.5.3.1. Samples were gently washed three times with phenol red-free DMEM before treatment. Bundles were then incubated with the 73 μM H_2_O_2_ solution for 1 h at 37 °C. After exposure, samples were washed three times with phenol red-free DMEM to remove residual H_2_O_2_ and supplied with fresh Schwann cell medium. After 24 h of recovery, viability was quantified using PrestoBlue as described previously. For each group, viability was expressed relative to its own untreated control (e.g., PLA + H_2_O_2_ vs PLA control, PLA–PDA 8 h + H_2_O_2_ vs PLA–PDA 8 h control and PLA–PDA 48 h + H_2_O_2_ vs PLA–PDA 48 h control).

#### 2.5.4 Dorsal Root Ganglia Culture and Immunofluorescence

To assess the influence of PDA coating on neurite outgrowth, dorsal root ganglia (DRGs) were cultured on the surface of the microfiber bundles (*n* = 10). Prior to DRG seeding, microfiber bundles were sanitized and conditioned following the same protocol described for the Schwann cell assays. DRGs were isolated following a previously reported protocol.^33^ Briefly, embryonic day 8 (E8) fertilized eggs were disinfected with 70% ethanol and carefully opened. The embryos were transferred to a Petri dish containing wash medium composed of Ham’s F-12 (11765054, Thermo Fisher Scientific), 10 mM HEPES (1530080, Thermo Fisher Scientific), and 1% penicillin/streptomycin to remove residual yolk and extra-embryonic tissue. The embryos were subsequently decapitated, and visceral organs were removed to expose the spinal cord and peripheral DRGs. Under a stereomicroscope, DRGs were carefully excised using ultrafine forceps by gently detaching them from the spinal cord and transferred to a Petri dish containing fresh wash medium. Individual DRG explants were then placed at the center of each microfiber bundle and incubated for 2 min at 37 °C in a humidified atmosphere containing 5% CO_2_ to promote initial attachment. Following this initial attachment period, 1 mL of DRG culture medium—consisting of wash medium supplemented with 1% l-glutamine (25030024, Thermo Fisher Scientific), 1% N2 supplement (17502048, Thermo Fisher Scientific), and 5 ng/mL nerve growth factor (NGF; HZ-1222, Proteintech)—was added, and the samples were incubated for 1 h under the same conditions. An additional 2 mL of DRG culture medium was then added. DRGs were maintained at 37 °C and 5% CO_2_ for 5 days, with medium changes performed every 2 days.

To visualize neurite outgrowth and axonal sprouting in response to PDA-coated microfibers, immunofluorescent staining of neuronal neurofilaments was performed after 5 days of culture. Fixation and blocking procedures were carried out following the same protocol described for the Schwann cell assays. Samples were incubated overnight at 4 °C with a neuron-specific primary antibody against βIII-tubulin (MAB1195, R&D Systems) diluted 1:200 in blocking buffer. After primary antibody incubation, samples were washed three times with PBS and subsequently incubated with an Alexa Fluor 488 donkey anti-mouse secondary antibody (A11029, Thermo Fisher Scientific), diluted 1:200 in blocking buffer, for 2 h at room temperature. Cell nuclei were counterstained as described previously. Samples were then rinsed three times with PBS, mounted using Vectashield (Vector Laboratories), and imaged using a fluorescence microscope (Eclipse 80i, Nikon). At this stage, DRG adhesion to the different sample types was assessed. DRGs that remained attached to the microfiber substrates were recorded, and the percentage of successful adhesion was calculated for each condition.

For image analysis, DRG images were first stitched together and subsequently analyzed using ImageJ software to quantify neurite outgrowth and axonal sprouting, following previously described methods.^16^ Briefly, neurite extension was assessed by measuring the longest neurite on each side of the DRG explant, with the dimensions of the DRG body excluded from the measurements. The area occupied by sprouting axons was quantified by subtracting the area of the DRG body from the total area covered by the DRG explant. For this analysis, images were converted to 8-bit grayscale format, followed by thresholding to generate a binary mask from which the relevant areas were measured. Neurite directionality was quantified using the “Directionality” plugin in Fiji (ImageJ) applying the Fourier-based analysis. The center of the fitted Gaussian distribution was used as the dominant orientation angle.

### 2.6 Statistical Analysis

Results are expressed as mean ± standard deviation (SD). All cell culture assays were performed in three independent experiments (*N* = 3), each including five technical replicates per condition (*n* = 5), unless otherwise stated. In *superplot* representations, small points correspond to individual technical replicates, whereas larger points indicate the mean value of each independent experiment. Statistical analysis was performed using GraphPad Prism 9.4 Software. Data normality was assessed using the Shapiro–Wilk test. Depending on the experimental design, comparisons were conducted using Student’s *t*-test or one-way or two-way ANOVA, followed by Tukey’s post hoc test when appropriate. Statistical significance was defined as * *p* < 0.05; ** *p* < 0.01; *** *p* < 0.001; **** *p* < 0.0001.

## 3 Results and Discussion

### 3.1 Morphological and Physicochemical Characterization of Polydopamine-Coated Microfibers

Peripheral nerve repair remains challenging due to axonal misdirection and incomplete reconnection following injury.^1,2^ While aligned synthetic scaffolds such as PLA microfibers can provide structural guidance, they often lack bioactivity. Here, PLA microfibers were functionalized with polydopamine to introduce antioxidant and cell-interactive properties while maintaining aligned topographical cues. The resulting constructs were systematically characterized to assess their suitability for supporting nerve regeneration. Following the PDA coating process, the surface of the PLA microfibers underwent a change in color from white to dark brown (Figure 1A–C). The darkening of the fibers was accentuated as coating time increased (Figure 1B and C); this color change is typical of PDA coating and indicative of PDA deposition on the substrate. The orientation of microfibers is a key determinant in peripheral nerve repair, as aligned topographical cues effectively steer axonal extension.^7,16,33,34^ When fibers are arranged in parallel, they provide a physical template that guides regenerating axons, ultimately limiting unnecessary branching and improving the overall regeneration outcome.^8^ The directionality analysis yielded average orientation angles of 90.6 ± 2.5° for PLA fibers and 89.3 ± 0.8° and 89.4 ± 0.4 for PDA-coated ones, 8 and 48 h, respectively (Figure 1D–F). Since these values did not differ significantly, the coating procedure can be considered nondisruptive to fiber alignment. This high degree of order suggests that the microfiber scaffolds will provide an appropriate structural framework to channel axonal regeneration along the desired trajectory. HRFESEM images of the surface of uncoated (Figure 1G) revealed a smooth surface, in contrast to the rougher texture observed on PDA-modified microfibers (Figure 1H and I). Moreover, it can also be observed that PDA particles increase with increased coating time, as previously reported, due to polydopamine interparticle aggregation.^35,36^ The increased roughness was corroborated by AFM, which was employed to examine the surface topography to quantify changes in surface roughness induced by PDA deposition. Representative AFM height images revealed a smooth homogeneous surface of bare PLA (Figure 1J), whereas PDA-coated microfibers exhibited progressively rougher and more heterogeneous morphologies with increasing coating time (Figure 1K and L). Quantitative roughness analysis confirmed these observations, bare PLA exhibited low surface roughness, with an average roughness (Ra) of 6.7 ± 1.4 nm and a root-mean-square roughness (Rq) of 8.6 ± 1.7 nm. Following PDA deposition, a significant increase in surface roughness was observed. After 8 h, Ra increased to 55.5 ± 13.2 nm and Rq to 61.5 ± 10.3 nm. Extending the coating time to 48 h led to a further significant increase in roughness with Ra and Rq values of 138.0 ± 11.9 nm and 173.5 ± 14.8 nm, respectively. This increase in roughness compared to bare PLA indicates the formation of a granular PDA layer on its surface and the substantial roughening with PDA-coating time suggests continued PDA accumulation in accordance with previous findings due to the self-polymerization behavior of PDA.^35,36^ To complement the surface characterization, the surface wettability of the PDA-modified substrates was examined, as this physicochemical property plays a key role in early cell-scaffold interactions and is known to be influenced by PDA deposition.^37,38^ Because individual microfiber bundles do not support stable sessile droplet formation (the water droplet rapidly penetrates between the fibers), static contact-angle measurements were performed on solvent-cast PLA films processed under the same PDA-coating conditions (Supplementary Methods, Section S1). Bare PLA films displayed a high contact angle (89 ± 4°), confirming their hydrophobic character (Figure 1G–I inset and Supplementary Figure S1). PDA deposition substantially improved surface wettability, reducing the contact angle to 63 ± 3° after 8 h of coating and to 54 ± 2° after 48 h. The progressive decrease in contact angle with longer coating times is consistent with greater PDA accumulation and the increased presence of hydrophilic catechol and amine groups on the surface.^39^ In addition, an increase in surface roughness, as seen from the AFM results, can also explain the increase in wettability (decrease in water contact angle) by the PDA-coated samples.^40^

**Figure 1:**
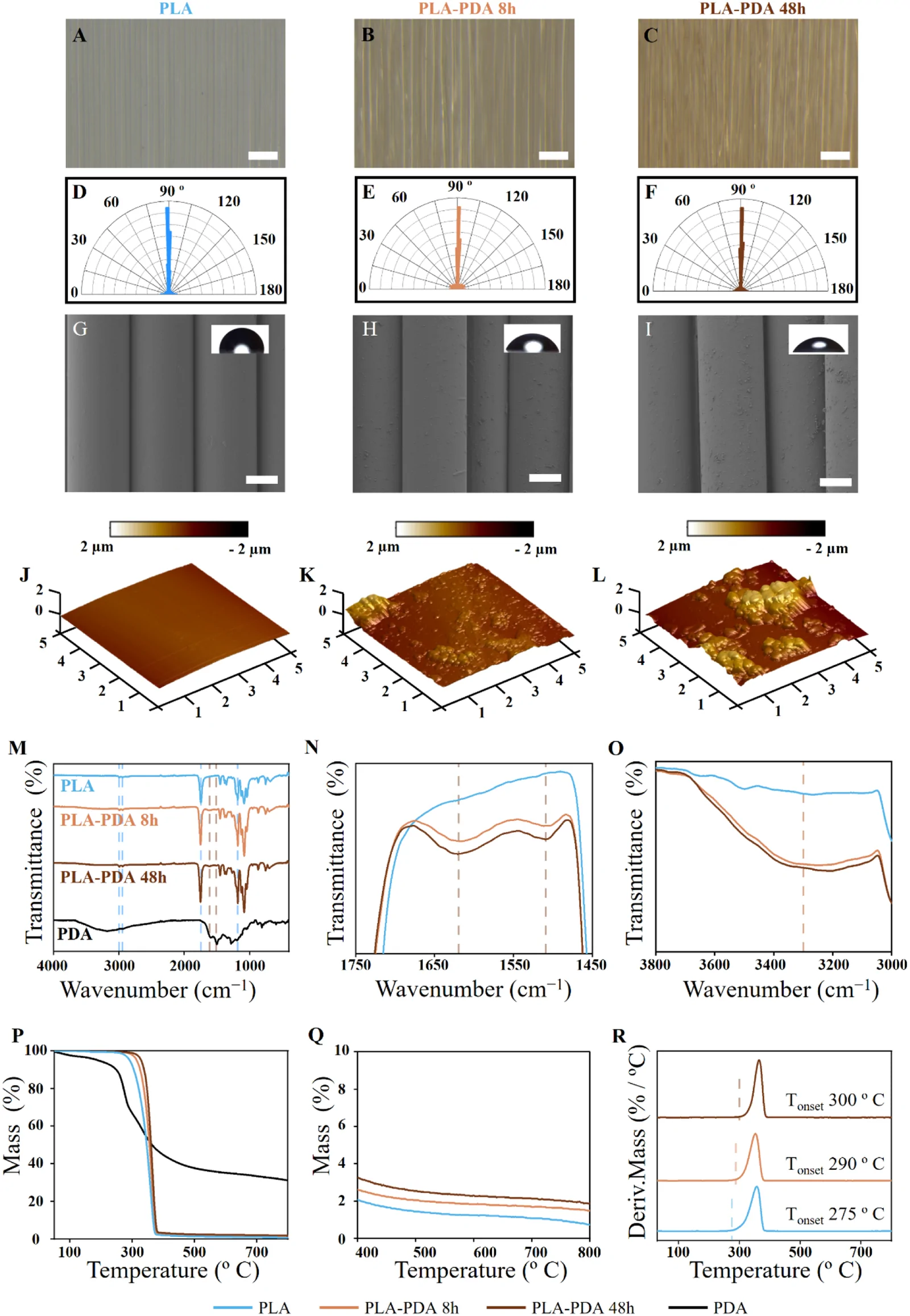
Morphological and physicochemical characterization of PDA-coated PLA microfibers. (A–C) Representative macroscopic images of aligned PLA microfibers before coating (PLA) and after PDA deposition for 8 h (PLA–PDA 8 h) and 48 h (PLA–PDA 48 h). Scale bar:100 μm. (D–F) Polar histograms showing fiber orientation of PLA, PLA–PDA 8 h, and PLA–PDA 48 h microfibers (*n* = 5). Statistical analysis was performed using one-way ANOVA. (G–I) HRFESEM images of PLA and PDA-coated microfibers, illustrating the surface morphology after 8 and 48 h of coating. Scale bar: 5 μm. Insets show representative static water contact angles measured on solvent-cast PLA films (*n* = 5) processed under identical PDA-coating conditions (see Supplementary Methods, Section S1). (J–L) Representative AFM height images of PLA, PLA–PDA 8 h, and PLA–PDA 48 h surfaces. All AFM height maps are displayed using the same z-range to facilitate direct comparison of surface roughness. (M) FTIR spectra of bare PLA (blue), PDA (black), and PDA-coated PLA (8 h, light brown; 48 h, dark brown) (*n* = 3). Blue dashed lines indicate characteristic PLA peaks, whereas brown dashed lines correspond to PDA-related bands. (N, O) Enlarged FTIR regions highlighting the C=C/N–H and O–H stretching bands, respectively. (P) Thermogravimetric analysis (TGA) curves of PLA, PDA, and PDA-coated PLA samples (*n* = 3; color coding as in panel M). (Q) Enlarged view of the residual mass region. (R) Derivative thermogravimetric (DTG) curves showing the degradation onset temperatures.

It should be noted that films and microfiber bundles differ in their macroscopic topography (flat versus curved architectures). Consequently, the contact angle measurements should be interpreted as a comparative assessment of the relative changes in surface wettability induced by PDA functionalization rather than absolute wettability values of the microfiber bundles. Although the measured contact angle is influenced by the macroscopic geometry of the substrate, the intrinsic surface chemistry is expected to be equivalent because both films and microfibers are composed of the same material and undergo identical PDA coating conditions. Therefore, solvent-cast PLA films provide a well-defined experimental configuration in which the effect of PDA functionalization on surface wettability can be interpreted without the additional complications introduced by the architecture of the microfiber bundles.

To further study the PDA coating onto the PLA microfibers, FTIR and TGA assays were carried out. The FTIR spectra (Figure 1M) of PLA microfibers exhibited all characteristic peaks of pristine PLA: 2997 cm^–1^ (−CH_3_ asymmetric stretching), 2948 cm^–1^ (−CH_3_ symmetric stretching), 1759 cm^–1^ (C=O stretching), 1184 cm^–1^ (C–O–C stretching), and 1092 cm^–1^ (C–O stretching).^41^ In the case of PDA (Figure 1M), characteristic peaks could also be detected: a broad peak around 3300 cm^–^1 (−OH stretching), 1598 cm^–1^ (N–H stretching) and characteristic peaks of the dihydroxyindole moiety at 1511 and 1279 cm^–1^ ^42,43^ which indicate a successful oxidative polymerization of dopamine into polydopamine.^44^ After PDA coating on PLA microfibers, additional absorption peaks at 1501 cm^–1^ and 1615 cm^–1^ appeared (Figure 1N and O). The absorption peak at 1615 cm^–1^ is attributed to the overlapping of the C=C stretching vibration in the aromatic ring and N–H bending vibration, and the peak at 1501 cm^–1^ can be ascribed to the N–H shearing vibration of PDA^45,46^ (Figure 1N). Additionally, a broad band around 3300 cm^–1^ (Figure 1O), assigned to hydroxyl groups, is also present in PDA-uncoated PLA microfibers.

The thermograms (Figure 1P) show that PLA microfibers undergo a single-step thermolysis process, consistent with previously reported findings^47^ due to thermal degradation of the polymer material. In the case of PDA, a three-step decomposition process is observed. First, an initial weight loss between 30 and 130 °C due to water evaporation. Second, a weight loss around 230 °C–330 °C, due to the degradation of the catechol moiety, and finally, a third decomposition step of the amine groups decomposed from 330 to 430 °C.^48^ For PLA–PDA fibers, the TGA curve largely followed the thermal behavior of uncoated PLA, but notable differences attributable to the PDA layer were present. First, the residual mass (Figure 1Q) was higher than that of bare PLA (Figure 1E), increasing from 0.6 ± 0.4% to 1.6 ± 0.1% and 2.0 ± 0.2% for the 8 and 48 h coatings, respectively, reflecting increased PDA deposition with longer coating times. Second, PDA surface coating enhanced the apparent thermal stability of the polymer. As shown in the derivative curves (Figure 1R), the degradation onset temperature (T_onset_) shifted from roughly 275 °C in PLA to about 290 and 300 °C after 8 and 48 h of coating, respectively. This trend agrees with previous studies reporting that PDA coatings can improve the apparent thermal stability of PLA-based materials.^49–51^ This effect may be attributed to the substantial PDA residue remaining on the microfiber surface at the onset of PLA degradation (Figure 1P), which may hinder the diffusion of volatile degradation products and thereby delay the apparent thermal decomposition of PLA. Nevertheless, it is worth noting that not all PDA modifications exhibit this effect; some studies have shown a decrease in T_onset_ relative to uncoated PLA, particularly at higher dopamine concentrations. This reduction is thought to arise from residual low-molecular-weight dopamine oligomers within the PDA layer, which can promote earlier degradation.^52^

### 3.2 In Vitro ROS Scavenging of PLA–PDA Microfibers

PDA is known to interact with reactive oxygen species through its catechol-rich structure, which provides redox-active sites (catechol, quinones, amine and imine groups) that can scavenge free radicals. These groups enable PDA to participate in hydrogen atom donation and electron transfer processes, allowing reactive species to be converted into more stable, less harmful products like water.^32,53,54^ Owing to these properties, PDA coatings are frequently evaluated for their capacity to mitigate oxidative stress. To assess whether PDA deposition conferred our PLA microfibers with antioxidant functionality, we performed ABTS and H_2_O_2_ clearance assays.

Uncoated PLA exhibited negligible antioxidant activity, removing only 3 ± 2% of ABTS•^+^ after 6 h (Figure 2A). In contrast, both PDA-coated groups showed a clear increase in radical scavenging. Within the first 30 min, PLA–PDA 8 h and PLA–PDA 48 h removed 6 ± 2% and 7 ± 2% of ABTS•^+^, respectively, and their activity continued to rise over time, reaching 30 ± 3% and 38 ± 2% at 6 h. The higher scavenging activity of the 48 h coating is consistent with its greater PDA deposition, as supported by TGA, and the resulting increase in the availability of redox-active groups. A comparable pattern was observed for H_2_O_2_ clearance (Figure 2B). PLA alone removed only 3 ± 1% of H_2_O_2_, whereas PDA-coated fibers achieved 11 ± 2% (8 h) and 19 ± 3% (48 h). Together, these assays demonstrate a coating-time-dependent relationship between PDA deposition and antioxidant functionality: increasing the coating duration from 8 to 48 h resulted in greater PDA deposition and correspondingly enhanced ABTS•^+^ and H_2_O_2_ scavenging.

**Figure 2:**
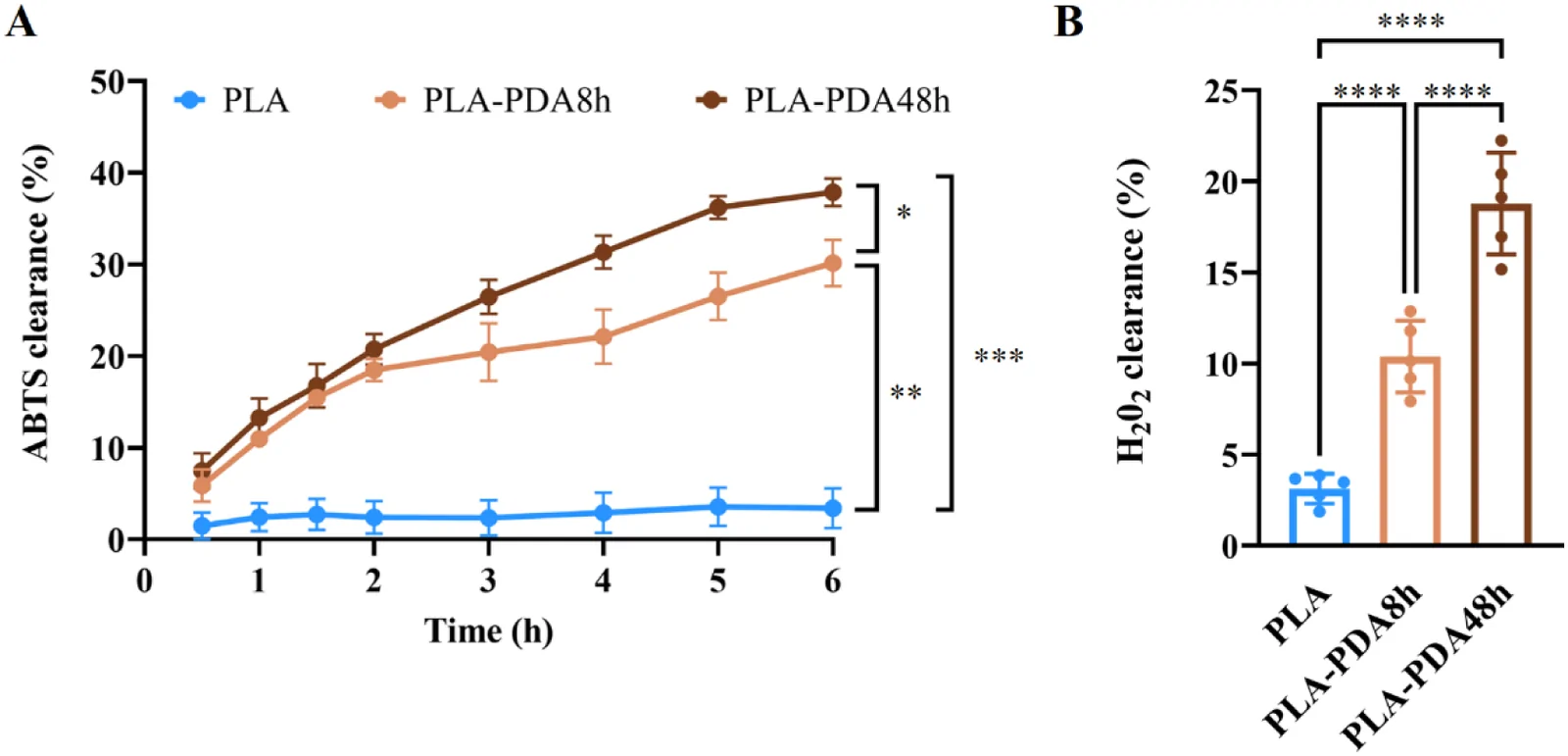
Antioxidant activity of PLA and PDA-coated microfibers. (A) Time-dependent ABTS•^+^ scavenging by uncoated PLA, PLA–PDA 8 h, and PLA–PDA 48 h microfibers over 6 h (*n* = 5). Statistical analysis was performed using two-way ANOVA. (B) Hydrogen peroxide (H_2_O_2_) clearance after 1 h (*n* = 5). Data were analyzed using one-way ANOVA. Data are presented as mean ± SD * *p* ≤ 0.05, ** *p* ≤ 0.01; *** *p* ≤ 0.001 and **** *p* ≤ 0.0001.

The ROS-scavenging properties of PDA have been widely reported, particularly for PDA nanoparticles (PDA-NPs)^55–59^ and PDA-NPs integrated into hydrogels, sponges, or other polymer matrices.^32,60,61^ These systems often report very high scavenging efficiencies, in some cases approaching 90% H_2_O_2_ removal.^32^ The lower values observed in our study are unsurprising when considering the different material architectures. Nanoparticles possess extremely high specific surface areas, maximizing the exposure of redox-active groups to the aqueous phase. Prior studies have shown that increasing PDA-NP size leads to reduced scavenging efficiency, reinforcing the importance of accessible surface area.^32,62^ In contrast, in our microfiber system, PDA forms a coating on a comparatively large-diameter substrate. Only the outermost PDA layer is fully exposed to the medium, while ROS-scavenging functional groups located in fiber–fiber contact zones are sterically hindered and likely less available for ROS interaction. This structural constraint naturally limits the bulk scavenging capacity of the material.

Importantly, for peripheral nerve repair, the moderate scavenging observed here may be advantageous rather than limiting. There is increasing recognition that ROS play a dual role in regeneration: while excessive ROS levels contribute to oxidative damage and impaired axonal regrowth, low to moderate ROS levels are essential for redox signaling, growth cone dynamics and axonal outgrowth.^30,63,64^ From this perspective, a scaffold that reduces oxidative stress without completely abolishing ROS may therefore be better suited to support regeneration. Importantly, ROS-scavenging activity alone does not provide the directional topographical guidance required to orient cellular and neurite growth. Thus, combining the structural guidance of PLA microfibers with the moderate antioxidant activity provided by PDA may create a balanced microenvironment conducive to nerve repair.

### 3.3 Behavior of hSCs on PLA–PDA Microfibers

In the peripheral nervous system, Schwann cells are key regulators of nerve regeneration, as they contribute to the formation of a microenvironment that supports axonal repair.^65^ Their ability to promote neural regeneration has been extensively demonstrated in both *in vitro* and *in vivo* models.^66–68^ Consequently, biomaterials intended for nerve repair must support Schwann cell adhesion and proliferation to facilitate axonal regrowth. At the same time, previous studies have reported cytotoxic effects associated with unpolymerized dopamine,^69,70^ highlighting the importance of assessing the biological response to PDA-based materials. For these reasons, the interaction of the fabricated microfibers with Schwann cells was systematically assessed *in vitro*.

To assess cytocompatibility, Schwann cell metabolic activity was quantified using the PrestoBlue assay (Figure 3A). PrestoBlue was selected because commonly used tetrazolium-based assays, such as MTS and CCK-8, were found to interact with the PDA coating (see Supplementary Methods, Section S2).

**Figure 3:**
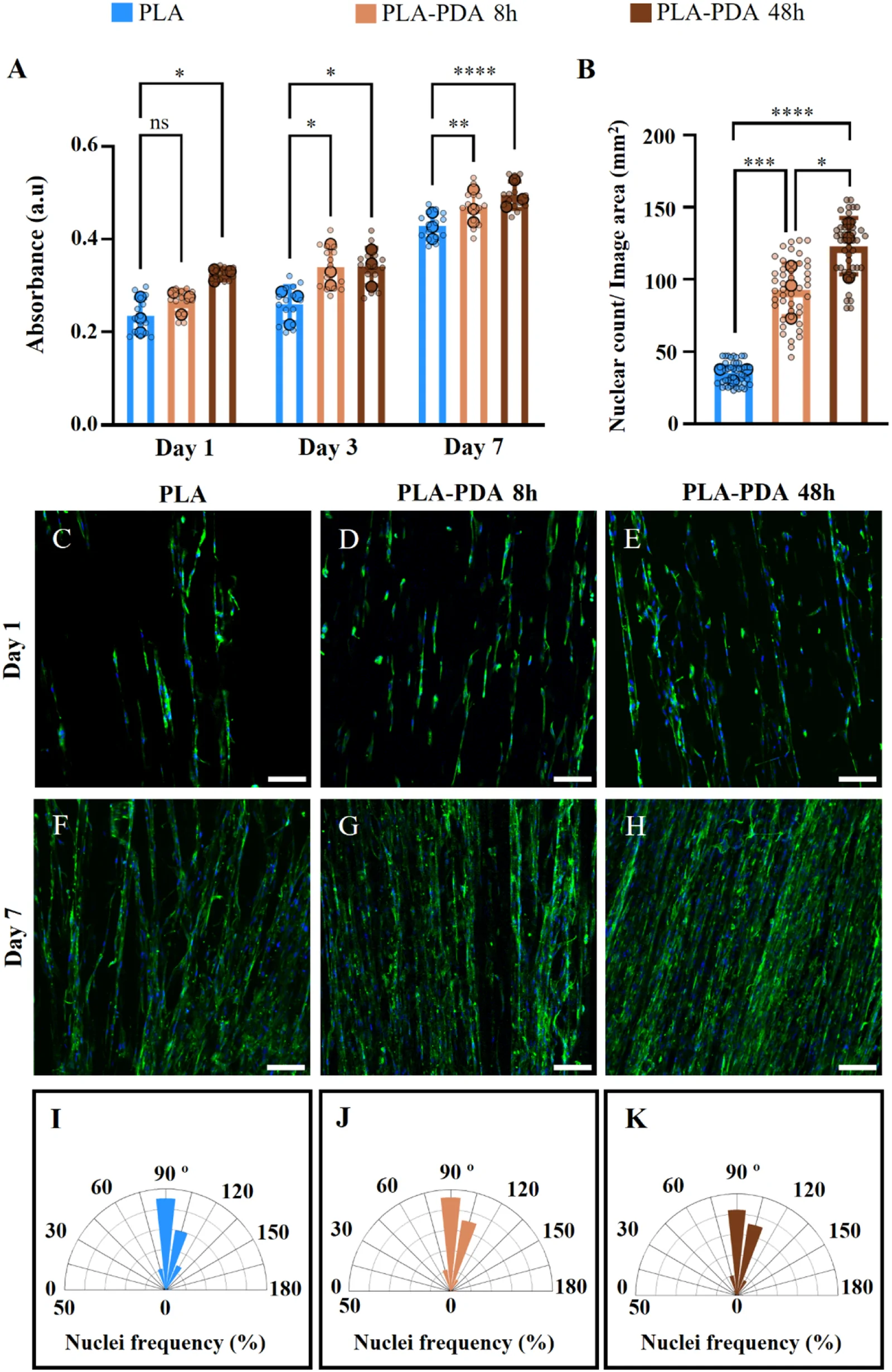
Schwann cell response to PLA and PDA-coated microfibers. (A) Metabolic activity of hSCs cultured on PLA, PLA–PDA 8 h, and PLA–PDA 48 h microfibers at days 1, 3, and 7, assessed by the PrestoBlue assay (*n* = 5 technical replicates per experiment; *N* = 3 independent experiments). Statistical analysis was performed using two-way ANOVA. (B) Quantification of hSCs nuclei after 1 day of culture (*n* = 5, *N* = 3). Statistical analysis was performed using one-way ANOVA. (C–H) Representative immunofluorescence images of hSCs seeded on PLA and PDA-coated microfibers (8 and 48 h) after 1 and 7 days of culture. S100β is shown in green and nuclei (DAPI) in blue. Scale bar: 100 μm. (I–K) Nuclear orientation analysis of hSCs cultured on PLA, PLA–PDA 8 h, and PLA–PDA 48 h microfibers after 1 day (*n* = 5, *N* = 3). Statistical analysis was performed using one-way ANOVA. Data are presented as mean ± SD. Statistical significance: n.s., not significant; * *p* ≤ 0.05; ** *p* ≤ 0.01; *** *p* ≤ 0.001; **** *p* ≤ 0.0001; ns not significant.

The results revealed a progressive increase in absorbance over time across all experimental groups, indicating that none of the scaffolds exerted cytotoxic effects on hSCs. Notably, both PLA–PDA 8 h and PLA–PDA 48 h microfibers exhibited higher absorbance values than bare PLA from day 1 onward. Among the PDA-coated samples, the PLA–PDA 48 h group showed the highest metabolic activity, although the difference relative to PLA–PDA 8 h was not statistically significant. To further investigate cell distribution and morphology, immunofluorescence staining for the Schwann cell marker S100β was performed (Figure 3C–H). As early as 24 h after seeding, hSCs displayed a characteristic spindle-like morphology aligned parallel to the microfiber axis. By day 7, the cells extensively covered the microfibers, forming a highly aligned cellular tapestry. This organization resembles the formation of Büngner bands, longitudinal Schwann cell strands that arise during the early stages of nerve repair and provide guidance for axonal extension from the proximal to the distal stump,^71^ which allows the guided elongation of growth cones from the proximal end to the distal end. Reproducing such aligned architectures *in vitro* has been shown to enhance axonal elongation by promoting directed growth and reducing delays associated with random or misoriented extension.^7,8,72^

Quantification of nuclear number after 1 day culture (Figure 3B), was consistent with the metabolic activity data. The PLA–PDA 48 h group exhibited the highest number of hSCs nuclei (124 ± 21 nuc.count/image area mm^2^), followed by PLA–PDA 8 h (92 ± 21 nuc.count/image area mm^2^) and bare PLA (37 ± 9 nuc.count/image area mm^2^). This trend is likely attributable to improved initial cell attachment on PDA-coated scaffolds, as previously reported for PDA-modified substrates.^73,74^ The elevated metabolic activity detected by the PrestoBlue assay can therefore be explained, at least in part, by enhanced early cell adhesion on PDA-coated microfibers. Analysis of nuclear orientation after 1 day of culture (Figure 3I–J), further corroborated the qualitative observations from immunofluorescence imaging. No statistically significant differences were detected in nuclear alignment among the different groups, with mean orientation angles of 93 ± 13°, 90 ± 12°, and 90 ± 14° for PLA, PLA–PDA 8 h, and PLA–PDA 48 h, respectively. These results indicate that PDA functionalization does not interfere with the intrinsic topographical guidance provided by the aligned microfibers.

Overall, these findings demonstrate that PDA-coated PLA microfibers support strong Schwann cell attachment while preserving the directional cues required for guided growth. The limited cell adhesion typically observed on PLA can be attributed to the absence of intrinsic cell-recognition motifs on its surface, which restricts early attachment.^75^ Although PDA is not a native extracellular matrix component and lacks classical integrin-binding domains, it introduces catechol and amine functional groups that enhance surface hydrophilicity and roughness, these surface modifications promote protein adsorption and provide anchoring sites that facilitate cell attachment to substrates.^76^ Moreover, the increased nanoscale roughness of PLA–PDA samples, as evidenced by the contact angle and AFM analyses (Figure 1G–I and J–L, respectively), has been widely associated with improved cell adhesion on polymeric substrates.^77,78^ Notably, PDA functionalization enhanced Schwann cell attachment without adversely affecting cell viability or the topographical guidance provided by the aligned microfibers.

### 3.4 In Vitro Oxidative Stress Protection of PLA–PDA Microfibers

To establish appropriate conditions for inducing oxidative stress, hSCs were exposed to increasing concentrations of H_2_O_2_ (0–1000 μM), and cell viability was assessed using the PrestoBlue assay. H_2_O_2_ caused a concentration-dependent reduction in hSCs’ viability (Figure 4A), fitting the corresponding dose–response curve revealed a half maximal inhibitory concentration (IC_50_) of 73 ± 3 μM (Figure 4B). Based on these results, 73 μM H_2_O_2_ was selected as the injury dose for subsequent experiments to model oxidative stress in hSCs seeded on microfibers.

**Figure 4:**
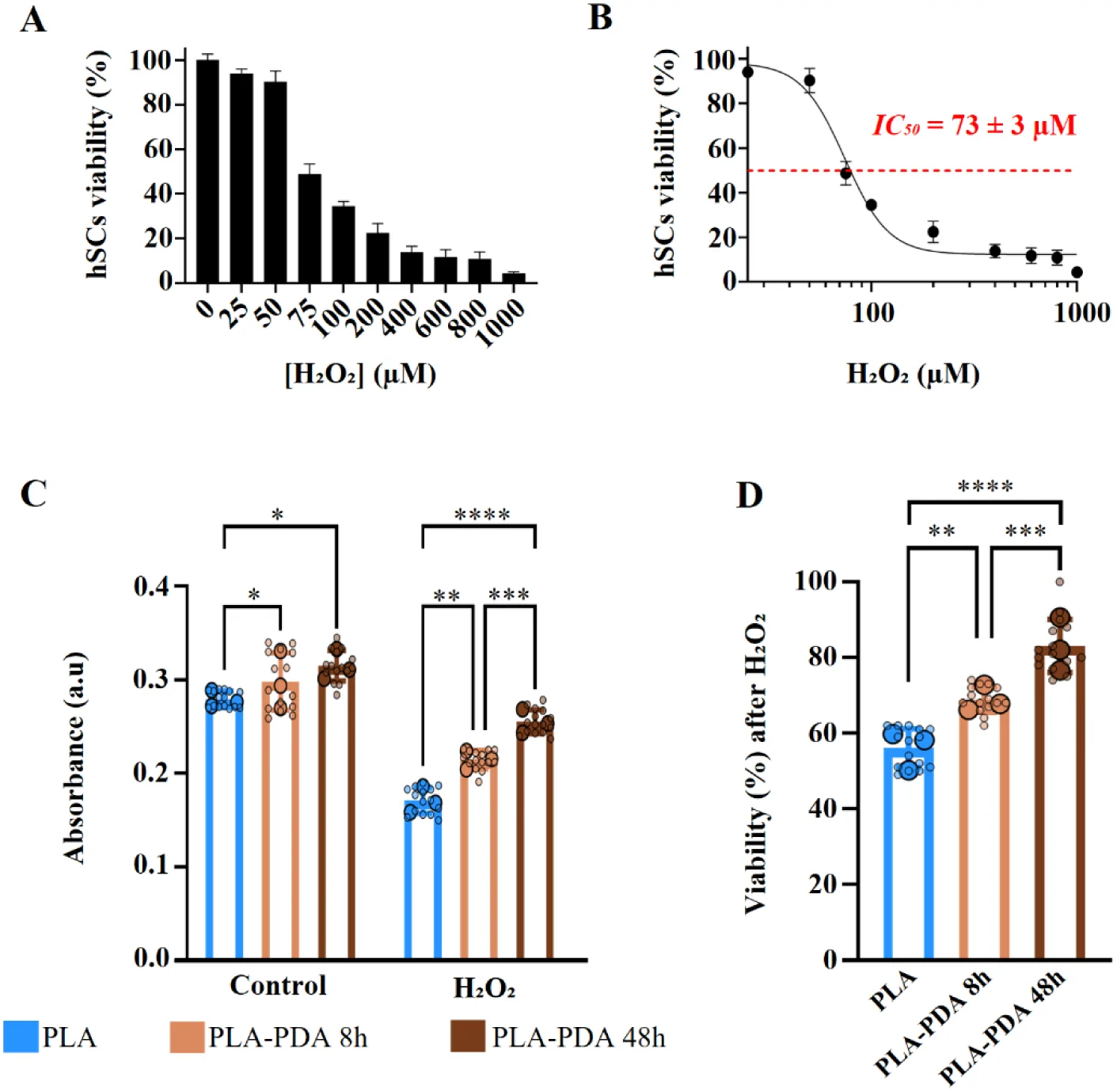
*In vitro* evaluation of H_2_O_2_stress tolerance on PLA and PDA-coated microfibers. (A) Viability of hSCs following exposure to increasing concentrations of H_2_O_2_ (0–1000 μM) for 1 h, with viability assessed after a 24 h recovery period and expressed as a percentage relative to untreated controls (*n* = 5, *N* = 3). (B) Corresponding dose–response curve fitted using a four-parameter logistic model, with H_2_O_2_ concentration plotted on a logarithmic scale. The red dashed line denotes 50% viability; the calculated half-maximal inhibitory concentration (IC_50_) was 73 ± 3 μM. (C) Absorbance values of hSCs cultured on unmodified PLA and PDA-coated microfibers, with or without exposure to 73 μM H_2_O_2_ for 1 h, followed by a 24 h recovery period (*n* = 5, *N* = 3). Statistical analysis was performed using two-way ANOVA. (D) Cell viability after 73 μM H_2_O_2_ exposure, calculated from absorbance measurements following the recovery period (*n* = 5, *N* = 3). Statistical analysis was performed using one-way ANOVA. Data are presented as mean ± SD. Statistical significance: * *p* ≤ 0.05; ** *p* ≤ 0.01; *** *p* ≤ 0.001; **** *p* ≤ 0.0001.

Following exposure to 73 μM H_2_O_2_, a decrease in absorbance was observed in all experimental groups cultured on microfibers compared with their respective untreated controls (Figure 4C), consistent with oxidative stress-induced cellular damage. However, the reduction in metabolic activity was markedly less pronounced for PDA-coated samples than for bare PLA microfibers. When cell viability was calculated relative to the corresponding control conditions (Figure 4D), hSCs viability on PLA microfibers was 56 ± 5%, whereas significantly higher viability values were obtained from PDA-coated substrates, reaching 69 ± 4% for PLA–PDA 8 h and 83 ± 9% for PLA–PDA 48 h. The enhanced tolerance to oxidative stress observed in PDA-coated microfibers is consistent with their antioxidative activity and H_2_O_2_ clearance. In particular, the superior performance of the PLA–PDA 48 h group likely reflects the higher PDA deposition achieved with longer coating times, which results in increased availability of redox-active groups and improved H_2_O_2_ scavenging capacity, as demonstrated in the ABTS and H_2_O_2_ clearance assays (Figure 2A and B). Together, these results establish a coating–property–function relationship in which greater PDA deposition is associated with enhanced antioxidant activity and, subsequently, improved Schwann cell tolerance to H_2_O_2_-induced oxidative stress. Importantly, PDA functionalization did not compromise the directional cellular organization imposed by the aligned microfiber architecture, as demonstrated by the alignment results described above.

While the present study focuses on Schwann cell viability, morphology, alignment, and tolerance to oxidative stress as indicators of scaffold cytocompatibility and functionality, further molecular analyses will be required to elucidate the mechanisms underlying the cellular responses observed on PDA-functionalized microfibers.

### 3.5 Effect of PDA on Neurite Outgrowth

Based on the enhanced Schwann cell response and antioxidant performance observed for the PDA-48 h coating, this condition was selected as a representative PDA-functionalized scaffold for a proof-of-concept assessment of its effect on DRG neurite outgrowth. Neurite extension was evaluated as a functional indicator of scaffold performance relevant to nerve regeneration. DRG neurofilaments were immunolabeled and visualized after 5 days of culture to assess neurite outgrowth on the different substrates (Figure 5A and B). PDA functionalization significantly improved DRG adhesion. Bare PLA microfibers exhibited an attachment rate of 60 ± 16%, which significantly increased to 92 ± 11% following PDA coating. These findings indicate that PDA-coated PLA microfibers possess enhanced cellular affinity and effectively support DRG attachment. This improvement can be attributed to mechanisms similar to those observed for Schwann cells, as the poor cell adhesion typically associated with PLA surfaces arises from the absence of natural cell-recognition sites. In contrast, the PDA coating introduces catechol- and amine-rich functional groups that increase surface hydrophilicity and roughness while providing anchoring sites for endogenous protein adsorption, collectively promoting improved cell attachment.

**Figure 5:**
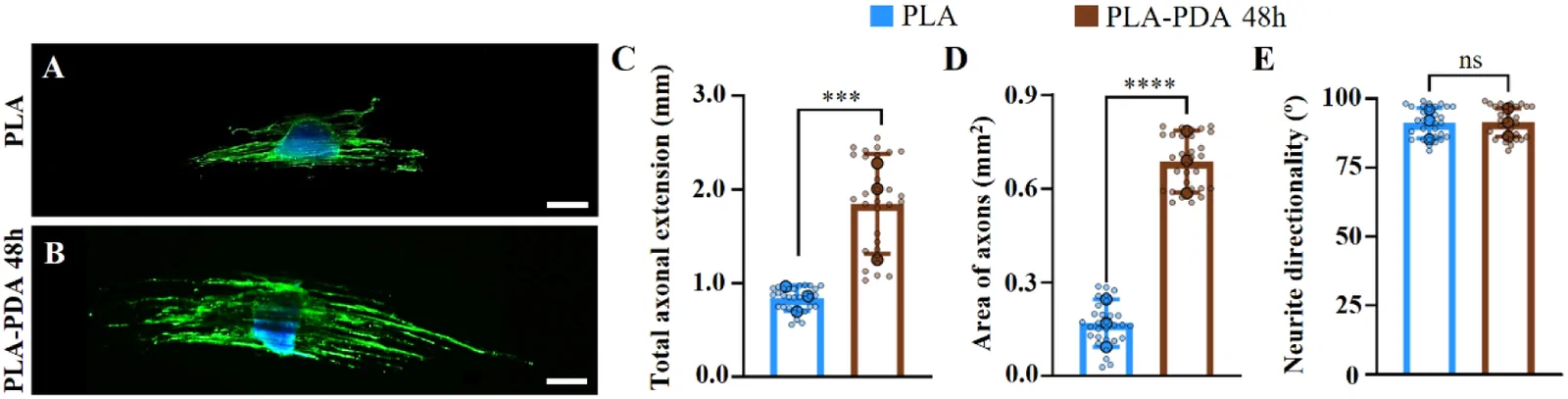
Effect of polydopamine coating on dorsal root ganglion (DRG) neurite outgrowth. Representative immunofluorescence images of DRGs cultured on (A) PLA and (B) PLA–PDA 48 h microfibrillar scaffolds for 5 days. βIII-tubulin–positive neurites are shown in green and nuclei (DAPI) in blue. Scale bar: 200 μm. (C) Total axonal extension was quantified by summing neurite outgrowth on both sides of the DRG explant (*n* = 10, *N* = 3). (D) Axonal area, calculated as the total DRG area minus the area of the DRG body (*n* = 10, *N* = 3). (E) DRGs’ neurite directionality (*n* = 10, *N* = 3). Statistical analysis was performed using a Student’s *t-*test. Data are presented as mean ± SD. Statistical significance: *** *p* ≤ 0.001; **** *p* ≤ 0.0001; ns not significant.

Quantitative analysis revealed that neurite extension was shortest on bare PLA microfibers (0.86 ± 0.13 mm) and significantly increased on PLA–PDA 48 h substrates (1.86 ± 0.61 mm), corresponding to an approximately 2-fold enhancement (Figure 5C). Similarly, the neurite-covered area was markedly greater on PLA–PDA 48 h microfibers (0.71 ± 0.12 mm^2^) than on bare PLA (0.17 ± 0.10 mm^2^), representing an almost 4-fold increase (Figure 5D). Axonal area reflects the extent of axonal sprouting and correlates with the number of neurites emerging from the DRG body, a critical factor for effective nerve regeneration.^33,79^ The enhanced neurite response likely reflects the combined surface changes introduced by PDA functionalization, including increased wettability, nanoscale roughness, and the presence of catechol- and amine-rich functional groups that can promote protein adsorption and cellular interactions. Increased micro- and nanoscale surface features have also previously been associated with enhanced neurite outgrowth on polymeric substrates.^80,81^

Despite the significant increase in surface roughness following PDA deposition, shown by the AFM results, no differences in neurite directionality were observed between groups, with both PLA and PDA-coated microfibers (Figure 5E) exhibiting a dominant neurite orientation of 91 ± 6° and 91 ± 7° degrees of alignment, respectively. This suggests that neurite orientation was primarily governed by the aligned microfiber architecture rather than nanoscale changes in surface topography introduced by the PDA coating. Thus, PDA functionalization enhanced neurite extension and coverage without compromising the directional guidance provided by microfiber alignment. This behavior is consistent with the response observed for Schwann cells and highlights the effective topographical guidance provided by the aligned microfiber architecture.

Taken together, these findings demonstrate the complementary functions provided by the two components of the system. The aligned PLA microfiber architecture provides a structural cue that governs directional cellular and neurite organization, whereas PDA functionalization introduces surface bioactivity and antioxidant functionality. Importantly, increasing PDA deposition enhanced antioxidant performance and Schwann cell tolerance to oxidative stress, while the selected PDA-48 h condition also promoted DRG attachment and neurite outgrowth without disrupting the directional guidance imposed by the aligned architecture. This integration therefore provides a comparatively simple approach for combining structural guidance with intrinsic surface and antioxidant functionality without requiring the incorporation of additional growth factors or conductive components. In contrast to strategies in which PDA serves as an interface for the immobilization or delivery of neurotrophic factors within multifunctional systems incorporating additional regenerative cues, the present approach directly exploits the surface bioactivity and intrinsic antioxidant properties of the PDA coating itself.

While the present study demonstrates promising effects of PDA functionalization on Schwann cell responses under H_2_O_2_-induced oxidative stress and DRG neurite outgrowth, the absence of *in vivo* validation represents a limitation when considering the translational relevance of these findings. Further investigation in appropriate peripheral nerve injury models will enable evaluation of the effects of PDA-functionalized aligned PLA microfibers on axonal regeneration and functional recovery. Such investigations represent an important next step toward establishing the regenerative potential and translational relevance of this approach.

## 4 Conclusions

Achieving effective peripheral nerve regeneration requires biomaterial strategies that incorporate key structural and biochemical cues relevant to nerve repair. Regenerative platforms must not only provide aligned architectures to prevent axonal misdirection but also actively support cellular function within the hostile post-injury microenvironment. In this work, aligned polylactic acid microfibers were functionalized with polydopamine to introduce bioactive and antioxidant properties while preserving their topographical guidance properties. We demonstrate that PDA deposition results in surface modification, leading to increased surface roughness and hydrophilicity, enhanced Schwann cell attachment, and improved cellular tolerance to oxidative stress. Importantly, increasing the PDA coating duration resulted in greater PDA deposition and antioxidant activity, which was associated with improved Schwann cell tolerance to H_2_O_2_-induced oxidative stress, establishing a coating–property–function relationship between PDA functionalization and biological response. PDA functionalization did not interfere with the intrinsic alignment cues of the microfiber scaffolds, allowing Schwann cells to maintain a highly oriented morphology along the fiber axis. While surface topography alone contributes to cell adhesion, our findings highlight that the addition of a bioactive PDA layer provides further functional benefits by mitigating oxidative stress under the conditions evaluated here. In addition, PDA-coated aligned PLA microfibers promoted dorsal root ganglion adhesion and neurite outgrowth while preserving directional guidance, further supporting their relevance as internal guidance substrates for nerve regeneration. Together, these findings demonstrate the complementary functions of the two components: the aligned microfiber architecture provides structural guidance, whereas PDA introduces surface bioactivity and antioxidant functionality. This comparatively simple approach combines directional guidance and intrinsic antioxidant functionality without requiring additional growth factors or conductive components. Overall, the PDA-coated PLA microfibers developed in this study represent a promising platform for nerve repair applications, combining structural guidance with biochemical protection. These findings provide a strong foundation for future *in vivo* investigations to determine whether the observed effects translate into enhanced nerve regeneration and functional recovery.

## Supplementary Material



## Data Availability

Data will be made available on request.

## Notes

File containing water contact angle results and viability kits (PDF)

## Abbreviations

ABTS: 2,2-azino-bis­(3-ethylbenzothiazoline-6-sulfonic acid)
BCA: bicinchoninic acid
DMEM: Dulbecco’s modified Eagle’s medium
DRGs: dorsal root ganglia
FBS: fetal bovine serum
FESEM: field emission scanning electron Microscopy
FTIR: Fourier transform infrared spectroscopy
hSCs: human Schwann cells
IC50: half-maximal inhibitory concentration
NGS: normal goat serum
PBS: phosphate-buffered saline
PDA: polydopamine
PFA: paraformaldehyde
PLA: polylactic acid
PNIs: peripheral nerve injuries
TGA: thermogravimetric analysis
