# Supplementary material for: Polydopamine Functionalization of Aligned Polylactic Acid Microfibers for Enhanced Neural Cell Interaction and Oxidative Stress Mitigation
Source: ACS Omega. 2026 Oct 28;11(44):68665–78. doi: 10.1021/acsomega.6c07293 (PMC13756715; doi:10.1021/acsomega.6c07293)
Supplement: Supplementary file 1 [file ao6c07293_si_001.pdf]

Supplementary Information

Polydopamine functionalization of aligned polylactic acid microfibers for enhanced neural cell interaction and oxidative stress mitigation

Authors: Francisco Navarro-Páez [a,], Fernando Gisbert Roca [a], Manuel Monleón Pradas [a,c]*

[a] Center for Biomaterials and Tissue Engineering, Universitat Politècnica de València, C. de Vera s/n, 46022, Valencia, Spain.

[c] Networking Research Center on Bioengineering, Biomaterials and Nanomedicine, Valencia, Spain

* Corresponding author: Francisco Navarro-Páez. e-mail: fnavpae@upvnet.upv.es

1. Water contact angle

Direct contact-angle measurements, to assess hydrophilicity, were not feasible on the microfiber bundles because the water droplet immediately seeped between the fibres. To overcome this limitation, PLA films were prepared by solvent casting and subjected to the same PDA coating conditions and procedures as the microfibers. Solvent-cast PLA films were obtained as described in[1]. Briefly, PLA pellets (Goodfellow, ME346310) were first dissolved in chloroform (Scharlab, CL02002500) at 1% wt/wt and stirred overnight at room temperature. The resulting solution was then poured into glass Petri dishes and left to air-dry for one week. After drying, the films were detached from the dishes and placed under vacuum at 70 °C for 24 h to remove any remaining solvent. Then the PLA films were die-cut using a biopsy punch (BP-80F, Kai Medical) to 8 mm discs and subjected to the same PDA coating conditions as microfibers. Water contact angle was measured using a Contact Angle Goniometer (L2004A1, Ossila) using the sessile drop method. A 5µL Milli-Q water droplet was placed in the sample (n = 5) and photographed and analysed using the Ossila software (Ossila Contact Angle, v.4.1.5). Data were expressed as mean ± standard deviation (SD).

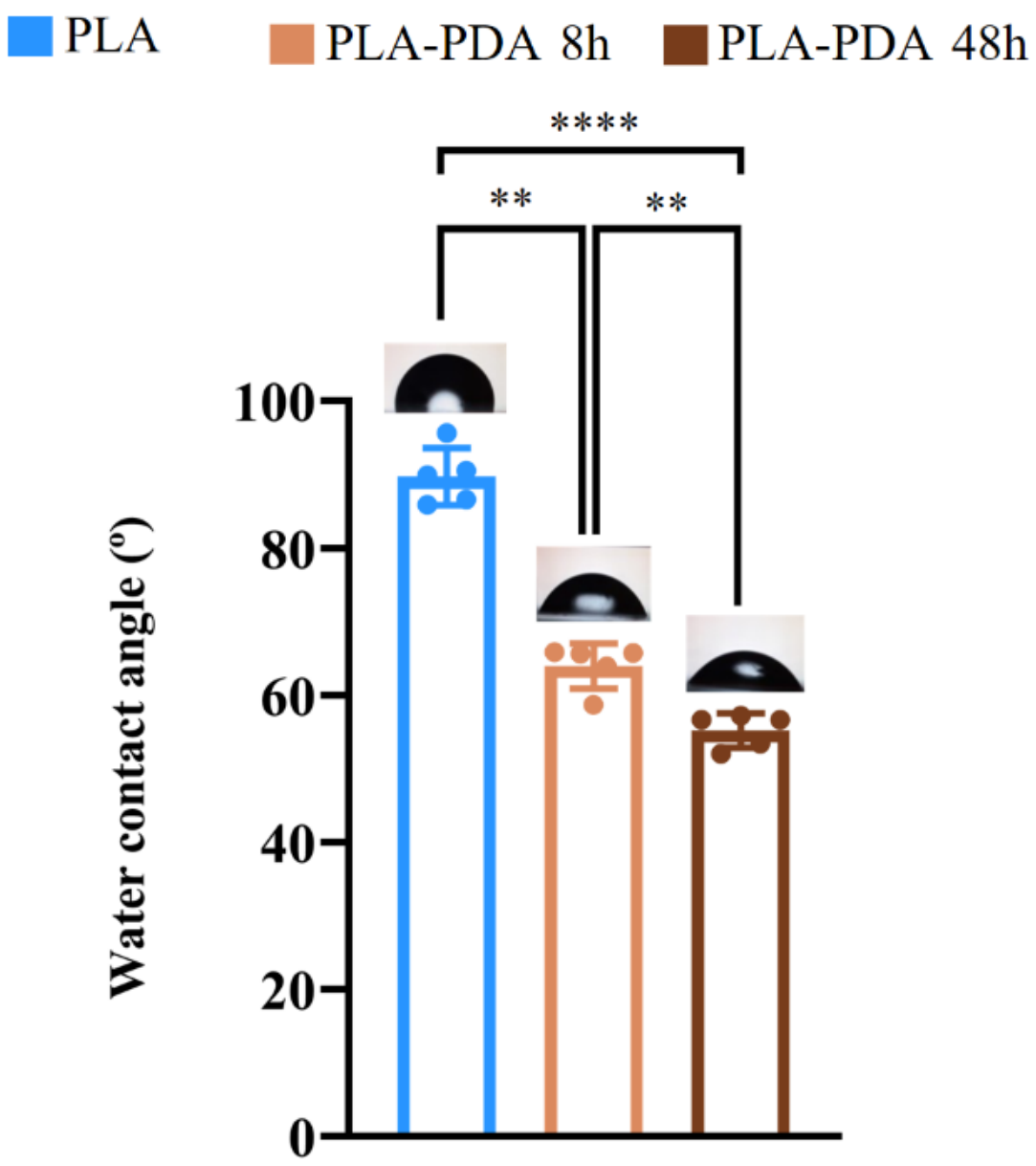

Figure S1. Water contact angle of PLA films before and after PDA coating. Representative static water contact angles measured on solvent-cast PLA films (n = 5), untreated (PLA) or coated with polydopamine for 8 h (PLA-PDA 8h) and 48 h (PLA-PDA 48h). Insets show representative droplet profiles. Data represent mean ± SD. ** $p \leq 0.01$ and **** $p \leq 0.0001$.

Surface wettability is known to influence how cells interact with a scaffold, and in many polymer-based scaffolds, a more hydrophilic surface favors early attachment[2,3]. As expected for PLA, the solvent-cast films showed a high-water contact angle (89 ± 4°), confirming their hydrophobic nature (Figure S1). After 8 h of PDA deposition, the contact angle significantly decreased to 63 ± 3° and extending the coating time to 48 h lowered it even further to 54 ± 2°. This shift toward higher wettability aligns well with the idea that longer coating times allow more PDA to deposit on the surface, increasing the presence of catechol and amine groups that interact with water[4]. Overall, the water contact angle measurements in PLA films indicate that PDA effectively modifies PLA surfaces' hydrophobicity.

It should be noted that films and microfiber bundles differ in their macroscopic topography (flat versus curved architectures). Consequently, the contact angle measurements should be interpreted as a comparative assessment of the relative changes in surface wettability induced by PDA functionalization rather than absolute wettability values of the microfiber bundles. Although the measured contact angle is influenced by the macroscopic geometry of the substrate, the intrinsic surface chemistry is expected to be equivalent because both films and microfibers are composed of the same material and undergo identical PDA coating conditions. Therefore, solvent-cast PLA films provide a well-defined experimental configuration in which the effect of PDA functionalization on surface wettability can be interpreted without the additional complications introduced by the architecture of the microfiber bundles.

2. Viability kits

To assess the viability of hSCs on uncoated and PDA-coated microfibers, it was first necessary to identify a metabolic assay that does not chemically interact with PDA. To this end, three commonly used viability reagents: MTS assay kit (ab197010, Abcam), CCK-8 assay kit (96992, Merck), and PrestoBlue™ HS Cell Viability Reagent (P50200, Thermo Fisher Scientific), were incubated with PLA-PDA 48h microfiber bundles ($n = 5$). Samples were sanitized and conditioned as described in Section 2*.5.1 Sample sanitization* and incubated, in the absence of cells, with each reagent diluted to 10%. After 4 h of incubation, absorbance was measured at the appropriate wavelength for each assay: 570 nm for MTS, 450 nm for CCK-8, and 570 nm with 600 nm as the reference wavelength for PrestoBlue. Data are reported as mean ± standard deviation (SD).

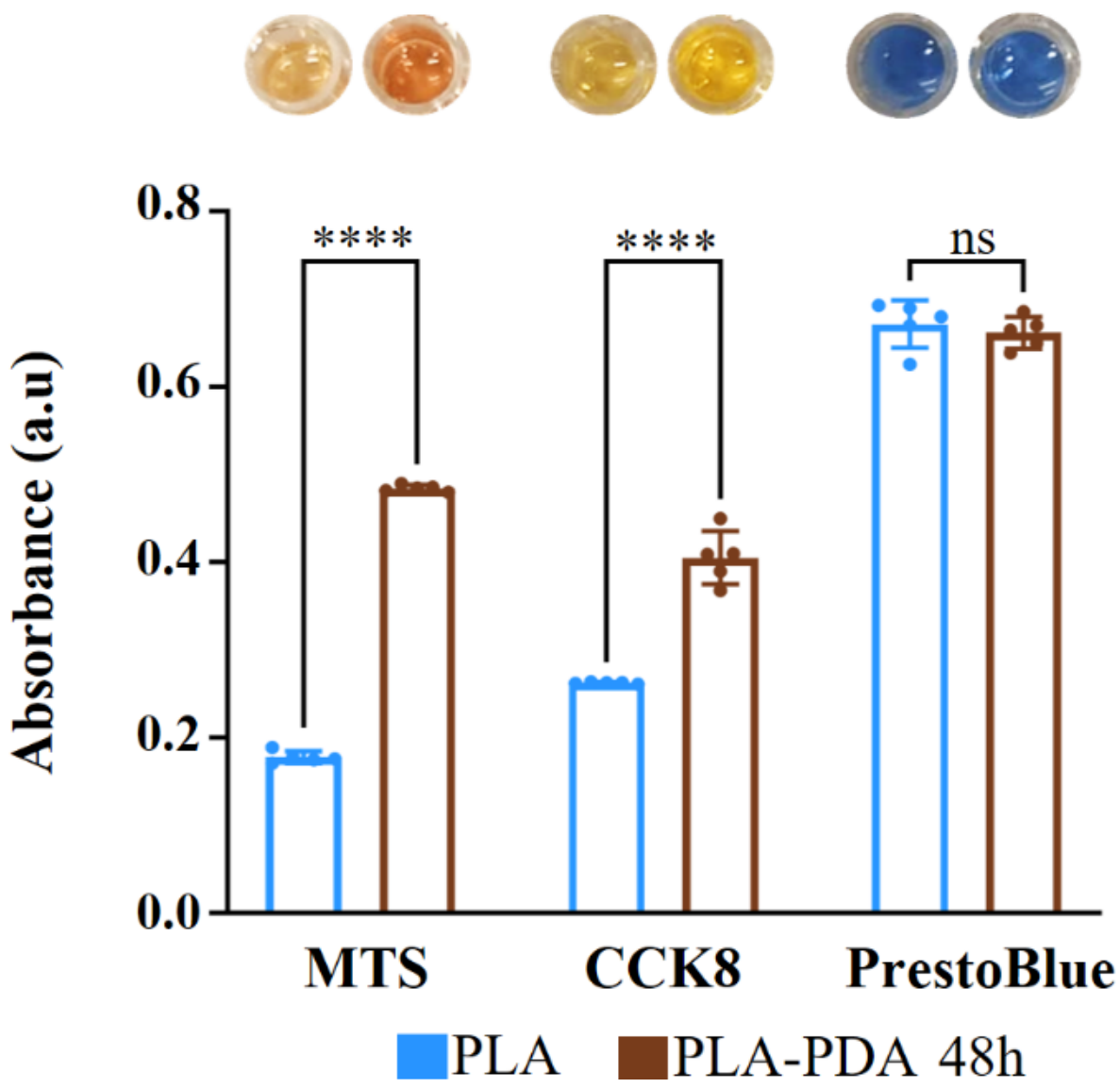

Figure S2. Interaction of PDA-coated microfibers with cell-viability reagents (MTS, CCK-8, and PrestoBlue). Corresponding absorbance signals of different available cell viability kits incubated with PLA and PLA-PDA 48h microfiber bundles (n = 5). Representative well images (top) show the colour development of MTS, CCK-8 and PrestoBlue reagents incubated in the absence of cells with uncoated PLA microfibers or PDA-coated PLA microfibers (PDA 48h). Data represent mean ± SD. **** $p \leq 0.0001$; ns not significant.

Following 4 h of incubation without cells, PLA-PDA48h samples exhibited markedly higher absorbance values in both the MTS and CCK-8 assays compared with unmodified PLA, whereas no significant difference was observed with PrestoBlue (Figure S2). In agreement with the quantitative measurements, wells containing MTS or CCK-8 showed a pronounced darkening when incubated with PLA-PDA samples, while PrestoBlue solutions retained a comparable blue coloration on both substrates.

These results indicate that PDA directly reduces tetrazolium-based reagents, specifically 3-(4,5-dimethylthiazol-2-yl)-5-(3-carboxymethoxyphenyl)-2-(4-sulfophenyl)-2H-tetrazolium in the case of MTS and 2-(2-methoxy-4-nitrophenyl)-3-(4-nitrophenyl)-5-(2,4-disulfophenyl)-2H-tetrazolium monosodium salt in the case of CCK-8. In contrast, the resazurin-based PrestoBlue assay was not significantly affected by the presence of PDA under identical conditions, suggesting that the resazurin/resorufin redox system is less susceptible to non-enzymatic reduction by the PDA coating on the microfibers. Even when material-only blanks are subtracted, PDA-mediated reduction can alter the kinetics of formazan formation, complicating the interpretation of the results, an issue that has also been reported for other PDA-based biomaterials[5]. On the other hand, the absence of a significant difference between PLA and PLA-PDA samples in the PrestoBlue assay supports the use of resazurin-based viability measurements for subsequent Schwann cell experiments on these scaffolds. By selecting a readout that shows minimal interaction with the PDA coating, scaffold-driven chemical artefacts are minimized, increasing confidence that changes in PrestoBlue signal in cell-seeded samples primarily reflect cellular metabolic activity. Collectively, these findings justify the use of PrestoBlue for quantitative viability assessment on PDA-modified microfibers and highlight the importance of verifying assay–material compatibility when working with redox-active coatings such as PDA.

REFERENCES

(1) Navarro-Páez, F.; Martínez-Férriz, A.; Martínez-Ramos, C.; Pradas, M. M. A Facile Method to Obtain Hydrophilic-Hydrophobic Hyaluronic Acid- Polylactic Acid Bilayer Films. *Surfaces and Interfaces* **2025**, *56*, 105590. https://doi.org/10.1016/j.surfin.2024.105590.

(2) Santos, L. M. S.; Oliveira, J. M.; Sonsin, A. F.; Nascimento, S. M. S.; Fonseca, V. M. L.; Silva, J. P.; Mendonça, C. R.; Jesus-Silva, A. J.; Barreto, E.; Fonseca, E. J. S. Physical and Chemical Modifications of Polymeric Surfaces to Enhance Epithelial Cell Adhesion. *ACS Omega* **2025**, *10* (44), 52703–52713. https://doi.org/10.1021/acsomega.5c06437.

(3) Bacakova, L.; Filova, E.; Parizek, M.; Ruml, T.; Svorcik, V. Modulation of Cell Adhesion, Proliferation and Differentiation on Materials Designed for Body Implants. *biotechnol. adv.* **2011**, *29* (6), 739–767. https://doi.org/10.1016/j.biotechadv.2011.06.004.

(4) Huang, Z.; Li, J.; Chen, X.; Yang, Q.; Zeng, X.; Bai, R.; Wang, L.; Huang, Z.; Li, J.; Chen, X.; Yang, Q.; Zeng, X.; Bai, R.; Wang, L. Photothermal Sensitive 3D Printed Biodegradable Polyester Scaffolds with Polydopamine Coating for Bone Tissue Engineering. *Polymers 2023, Vol. 15,* **2023**, *15* (2). https://doi.org/10.3390/polym15020381.

(5) Awasthi, A. K.; Gupta, S.; Namdev, K. R.; Banerjee, A.; Srivastava, A. Polydopamine and Dopamine Interfere with Tetrazolium-Based Cytotoxicity Assays and Produce Exaggerated Cytocompatibility Inferences. *Biomater. Sci.* **2021**, *9* (9), 3300–3305. https://doi.org/10.1039/d1bm00140j.
